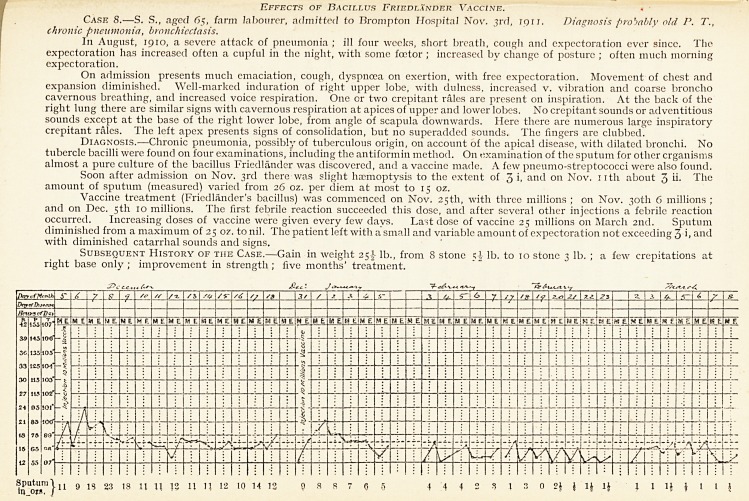# The Secondary Infections of Pulmonary Tuberculosis and Their Treatment by Vaccines
1Introduction to a discussion at a meeting of the Bristol Medico-Chirurgical Society on May 13th, 1914.


**Published:** 1914-06

**Authors:** S. H. Habershon

**Affiliations:** Physician to the Brompton Hospital for Consumption and Diseases of the Chest


*" Scire est nescire, nisi id me*
*Scire alius sciret."*

JUNE, 1914.

### THE SECONDARY INFECTIONS OF PULMONARY TUBERCULOSIS AND THEIR TREATMENT BY VACCINES.[1]

BY

S. H. HABERSHON, M.D. Cantab., F.R.C.P.,

*Physician to the Brompton Hospital for Consumption and Diseases of the Chest.*

CERTAIN constitutional types are, there is little doubt, peculiarly liable to contract tuberculosis. This susceptibility is usually spoken of as an inherited characteristic. But whether the individual is prone to such a tendency or whether he has a greater resisting power, tuberculous infection may be contracted in both classes and yet the disease itself remain latent.

There is ample evidence to prove that either a latent form of disease or an active and progressive tuberculosis render the individual also susceptible to invasion of other infective

[1] Introduction to a discussion at a meeting of the Bristol Medico-Chirurgical Society on May 13th, 1914.

organisms, and more especially to those of the catarrhal type.

The old-fashioned popular impression that pulmonary tuberculosis was often due to a " neglected cold " is in reality the expression of these facts now more scientifically explained. In the histories of our tuberculous patients we find the onset usually attributed to influenza (so called), or to some catarrhal affection, pneumonia, bronchitis, pleurisy or " feverish cold." In some cases, no doubt, the uncomplicated tuberculous invasion is ushered in by an attack simulating a febrile catarrh, but more often it is the catarrhal attack that awakes into activity a latent tuberculosis.

And if this is true of the onset of symptoms of pulmonary tuberculosis when the malady has previously been latent, it is still more true of the progressive disease. Activity is often enhanced and exaggerated by the prevalence of catarrhal affections due to the invasions of secondary organisms. Whether by the inflammatory action induced, or by the lowering of the vitality of the patient, these keep up the activity of the process and aggravate the symptoms.

It has always seemed to me that too little attention has been paid to the secondary complications of pulmonary tuberculosis, and that many of the more acute symptoms are not infrequently attributed to the tuberculous invasion when in reality they are due to the secondary infection. Not only may the symptoms of the pulmonary tuberculosis be masked by the secondary infection causing catarrhs of various kinds, but even the character of the catarrhal disease itself may be modified by the presence of tubercle. A special instance in point is seen in pneumonic tuberculosis.

The patient is seized with an attack simulating ordinary lobar pneumonia, though the severe constitutional symptoms (rigors and vomiting, etc.) are absent. No definite crisis occurs, but the temperature is kept up and assumes a hectic type, perhaps for weeks or months after the onset, while other unusual symptoms, such as late hæmoptysis, may supervene.

In most cases it is found that, in addition to the tubercle bacillus, Fraenkel's pneumococcus is also present.

The tuberculosis is masked by the pneumonia, and the character of the pneumonic attack is modified by the presence of the tubercle bacillus. I venture to give three instances of different character illustrating the methods in which *secondary infections* are often *the cause of an awakening of activity of a latent tuberculosis,* or at least of a tuberculosis not especially active.

**Case A.**—E. S., aged 45, a stonemason's foreman, was taken ill with all the signs of acute pneumonia. His family history was bad, and previous to the attack he had suffered from a rather persistent cough and loss of weight with malaise and debility. I was called into consultation because the pneumonic attack followed a rather unusual course, and at the end of a fortnight presented no crisis and developed a hectic type of temperature. A day or two previous to my visit there was a sharp attack of hæmoptysis. After several examinations of sputum T.B. were found, and in all a diplococcus pneumoniæ was present. The patient was eventually treated with Marmorek's serum, and after two months completely recovered, while the physical signs of consolidation of the right upper lobe entirely disappeared. The patient lost all cough and expectoration, and gained considerably in weight, while all T.B. disappeared from the sputum. He was perfectly well some five years after the attack, and I have lost sight of him since.

**Case B.**—A previous resident at the Brompton Hospital inoculated his finger with tubercle when engaged in pathological work. The result was a small nodule on one of the fingers below the nail (about the size of a small pea). This produced an intractable sore, and was excised by the surgeon to the Hospital. A microscopic examination proved the nodule to be tuberculous, with giant cells in the midst of granulation tissue. Six months later there was an epidemic of influenza in the Hospital, and the resident in question was taken ill with febrile disturbance, "the sensation of chills," headache and a definite bronchial catarrh. On the second or third day of the attack he observed some streaks of blood in the sputum, and a bacteriological examination of a small pellet of mucus proved it to be teeming with tubercle bacilli. Signs of disease at one apex developed.

The patient left the hospital and went to a sanatorium, returning in a year completely well. He has never had any trouble since and it is now some twelve to fifteen years ago.

**Case C** was of a different type. A young man of 19 with a desperate family history. The father and one sister were alive and well, but the mother died early from pulmonary tuberculosis, and some five brothers had all died between the ages of 18 and 25 from acute pulmonary tuberculosis. I was called to see the patient with an acute attack of catarrh, and found a bronchial attack with abundant crepitant sounds at the base of the lower lobe of the left lung. The temperature was raised, there were cough and expectoration, but no tubercle bacilli. In a few weeks the signs cleared up, and the young man on recovery was sent to an army coach in a healthy suburb of London contrary to my advice. I was again called to see him some three months later with a severe attack of hæmoptysis. Signs of disease were present at the apex of the left upper lobe, and he died six months later of acute pulmonary tuberculosis. This case occurred nearly twenty years ago.

I cite these three cases as instances of a secondary infection producing an exacerbation or an awakening into activity of a tuberculosis that in all probability was latent in each case.

I do not leave out of account a large number of cases of pulmonary tuberculosis that are uncomplicated and commence insidiously with cough, expectoration, night sweats and gradual development of symptoms due to infiltration of one or both apices, nor do I speak of cases that are also uncomplicated by secondary catarrhs and that commence with acute gastric or dyspeptic symptoms, or of further cases that may be ushered in by an attack of hæmoptysis, and this is the first warning of the onset of pulmonary tuberculosis.

None of the above examples of secondary infections at the onset need special vaccine treatment, and they are not the class of cases included in the scope of the present paper.

But when the lungs are invaded with tuberculous disease one of two processes occurs. In an individual with good resisting power the disease becomes quiescent and fibrous, and contraction and resolution occur. Or the disease may, in other cases, proceed to active and progressive extension. In both classes of cases there is a tendency to an invasion of the lungs by secondary infections and organisms.

The presence of other infective organisms than the tubercle bacillus in the sputum is no proof that the patient is suffering from a toxæmia due to any of these. Under normal conditions a variety of pathogenic bacteria are present in the mouth, and it is common to find the presence of staphylococci, pneumococci and streptococci in the mouths and nasal passages of healthy individuals.

Residence in a sanatorium often causes the disappearance of pathogenic organisms from the sputum, but in some cases they persist and are even capable of virulent infection of other individuals.

An excellent piece of work has been done on the subject of secondary infections by Dr. A. C. Inman,[1] the director of the Bacteriological Laboratories of the Brompton Hospital. As he states, "all exposed ulcerated surfaces are especially prone to secondary contamination with pathogenic organisms," and "ulcers of the lungs can therefore not be expected to escape pollution," and indeed the walls of tuberculous cavities have been shown (as he states) by Koch in 1884 to contain secondary bacteria.

We are therefore met with the following facts :—

The conditions of onset of pulmonary tuberculosis are frequently complicated by signs and symptoms demonstrating the simultaneous invasion of a secondary infective organism ; in most of the cases of acute and chronic pulmonary tuberculosis in which sputum is present we find abundant evidence of the presence of one or more pathogenic organisms ; in many of the cases of pulmonary tuberculosis with febrile disturbance and the presence of such bacteria in

[1] "A Contribution to the Study of Secondary Infections in Pulmonary Tuberculosis, *Lancet*, 1912, I. 975.

the sputum, we have to determine whether the signs and symptoms are due to the primary tuberculous disease or to the secondary pathogenic organisms, or to both, and on the other hand it is necessary to discover whether the patients are merely the carriers of such virulent organisms in the same manner in which individuals may be typhoid or diphtheria carriers, and not themselves sufferers from any form of toxæmia other than that derived from the tubercle bacillus.

Now the first mistake that is made in deciding what cases are suitable for vaccine treatment is one that I have myself made. In all my earlier cases I have concluded that if a pathogenic organism was found in abundance in the sputum, in addition to the tubercle bacillus, this was an adequate reason for the preparation of a vaccine and for treatment by its hypodermic injection. Even in some of the cases that I show and describe to-night I am not sure that I have not failed through want of more careful and thorough investigation.

How then can we determine whether the signs and symptoms of a tuberculous patient are due to the toxæmia of the tuberculous virus alone, or to that of a secondary pathogenic organism, or to both? For it is obvious that if the offending secondary organisms which are present in the sputum are not producing any toxæmia or symptoms, it is useless to prepare and inject a vaccine, and this is in fact what is often done, to the discredit of vaccine treatment.

I must candidly confess that though I feel I am now in a position to recommend the best and most favourable course to adopt, it is only in a few cases that I have learnt to follow this plan. Several methods of determination present themselves which guide us.

(*a*) *The physical signs.*

(*b*) *The symptoms*, including the character of sputum.

(*c*) *The bacteriological examination* of sputum.

(*d*) *The opsonic index* of the blood to tubercle and to each pathogenic organism.

I propose to deal with each of these methods as briefly as possible.

(*a*) *Physical signs.*—The prevalence of signs of catarrh—general rhonchus and sibilus, or, in addition to signs of consolidation at one or both apices, the presence of bronchitic râles, and above all of numerous basic crepitations. Occasionally signs of induration of lung or of pleural thickening at one or both bases may be present.

(*b*) *Symptoms.*—These have an intimate connection with physical signs. If the patient is not ill, and presents a condition of general well-being without much febrile disturbance, and yet there are universal crepitations or a large area of lung is affected with catarrhal signs, we may fairly conclude that these do not all represent softening tubercle. We are not wrong in attributing these moist sounds, if abundant, to a bronchitic condition.

If the temperature is raised and the case febrile, it becomes more difficult to assign the proper share to the tuberculous and the catarrhal infection. In such case we must investigate further.

The sputum of a case of tuberculosis of lungs in its softening stage is usually purulent and nummulated, and elastic tissue is often observed in cases of destructive ulceration. In catarrhal cases the sputum is often abundant and frothy, while it is rarely purulent, consisting mainly of mucous or mucopurulent secretion. This does not apply to the cases of tuberculous ulceration of larynx, where there is often profuse salivation and frothy sputum. But apart from general condition, febrile disturbance and appearance of sputum, it is common for tuberculous patients in whom the primary disease is quiescent or inactive to suffer from symptoms, not only of bronchitis with excessive cough and wheezing, but from paroxysmal asthma. Dyspnœa is a common occurrence, and especially of a spasmodic character in cases infected with the diplococcus pneumoniæ, and also with the micrococcus catarrhalis. As a rule, spasmodic asthma with a latent tuberculous focus in one or both lungs is due to the invasion of one or other of these two organisms. In one case it has occurred with a staphylococcus aureus, but this is rare and usually uncomplicated with tubercle.

(*c*) *Bacteriological examination* of the sputum for secondary organisms.—The following are the most common catarrhal and other pathogenic organisms found: (1) Micrococcus catarrhalis; (2) bacillus Friedländer; (3) Fraenkel's diplococcus pneumoniæ; (4) a diplo-streptococcus; (5) staphylococcus aureus, etc.; (6) a streptococcus; (7) bacillus coli communis (rare).

It is not always that a pure culture of one of these organisms is obtained. It is more common to find three or four present on the first culture medium with one in large preponderance. A sub-culture is then made from the one that is in excess, and this should first be investigated.

(*d*) The *test of the opsonic index.*—As Inman in his admirable paper states, in the opsonic index (discovered by the researches of Almroth Wright) we have a means of measuring a deficiency as well as an increased production of protective substances to the invasion of bacterial organisms. In the febrile conditions of pulmonary tuberculosis Inman established the inverse ratio of the opsonic index to the temperature, and when dealing with uncomplicated pulmonary tuberculosis, we can estimate its activity or the reverse by carefully watching this ratio.

Where a secondary complication is present no means other than the estimation of the opsonic index exists by which we can with certainty determine whether the febrile disturbance (if such is present) is due to the tuberculous toxæmia or to that of the secondary infection, or to both.

Therefore in all cases which seem to be suffering mainly from the results of secondary infections, though we may be largely guided by the physical signs and symptoms, and by the preliminary examination of sputum and the isolation of certain catarrhal and other pathogenic organisms, we are to some extent working in the dark when we are guided by these facts alone.

The investigation of the opsonic index first to tubercle and in turn to each of the other secondary bacteria is an elaborate one, and has been worked out by Inman and recorded in a few cases in the paper referred to. It is not feasible in the majority of cases, but in cases of doubt it is better to have the opsonic index to tubercle investigated even if this alone is possible. The difficulty of such a complicated research makes it necessary to rely in large measure on the first three considerations. But let me impress the fact that in a febrile case, or even in a case of pulmonary tuberculosis with wide oscillations of temperature which is not necessarily febrile, the inverse ratio between the opsonic index to tubercle and the temperature is an important aid to the differentiation between the toxic effect of the tuberculosis and that (if present) of the secondary organism.

The whole subject of the treatment of secondary infections is still in its infancy, and I have ventured to tell you plainly some of the reasons why I think the success of treatment in some of my cases at the Brompton Hospital has been extremely variable. And yet, as I have stated, to judge correctly whether the patient is suffering from the toxæmia of a particular secondary organism is not possible in the majority of cases because of the elaborate nature of the investigation. In most cases we must be guided by physical signs and symptoms, and if several organisms are present we should try in the first instance a vaccine prepared from the preponderating one. If this is not successful, and we are still convinced that the symptoms are not due to the tuberculosis, it will be necessary to have other vaccines prepared and try each in turn. When one pathogenic organism alone is present this difficulty does not arise.

The technique of the preparation of vaccines need not detain us. When the sputum is collected the mouth should first be rinsed with a dilute antiseptic solution. Formalin is the antiseptic employed at the Brompton Hospital. The early morning sputum should be obtained. A culture is made on agar or gelatine, and if necessary sub-cultures are made.

The methods of the injection of vaccines will be described in the narration of cases.

If there is reason to believe that the febrile disturbance is out of all proportion to the area and activity of disease as expressed by physical signs and tuberculo-opsonic index, the teeth should first be examined.

In hospital patients *pyorrhœa alveolaris* is a frequent cause of septic toxæmia, and the removal of exceptionally bad teeth or the treatment of others when pyorrhœa is present frequently has a marked effect. Before proceeding to vaccine treatment this must be carefully considered.

Two cases are given to demonstrate the improvement resulting from treatment for pyorrhœa. (Cases 1 and 2.)

CASE I. EFFECTS OF TOOTH EXTRACTION ON THE TEMPERATURE.

CASE I.—A. B., aged 21. Admitted Brompton Hospital November 1st. Chronic pulmonary tuberculosis, with pyorrhœa. Cough, loss of flesh, consolidation at right apex.

Opsonic index to tubercle: 1.28 before exercise, 1.43 after exercise; reaction after 1/5th mgm. of O. T. No T. B. found.

The erratic temperature was due to pyorrhœa alveolaris. General improvement occurred after extraction of teeth. Weight increased from 9 stone 1¼ lb. on Nov. 14th to 10 stone 1 lb. on Dec. 16th.

Case 2. Effects of Tooth Extraction on Temperature in a Case of Chronic Pulmonary Tuberculosis with Pyorrhœa.

Case 2.—T. C. C., aged 40. Admitted to Brompton Hospital June 21st, 1912. Bus conductor. Cough and expectoration 6 months. Pleurisy right side four weeks ago. Hæmoptysis in Jan., 1912. Physical signs of disease. Induration of right upper lobe with small apical cavity. Almost dry. A few inspiratory crepitant râles, disappearing after a few respirations. T. B. in sputum.

The erratic temperature was found to be due, not to the tuberculosis, but to pyorrhœa alveolaris.

The chart demonstrates the fall of temperature and diminished oscillation after tooth extraction.

Result: General improvement; diminution of cough and sputum; gain in weight of $3\frac{1}{2}$ lb.

CASE 2. EFFECTS OF TOOTH EXTRACTION ON TEMPERATURE IN A CASE OF CHRONIC PULMONARY TUBERCULOSIS WITH PYORRHŒA.

TEMPERATURE CHART SHOWING THE EFFECTS OF INJECTION OF A DOSE OF PNEUMOCOCCUS VACCINE.

CASE 3.—P. McD., æt. 35, November 1st, 1914.

It will be observed that the first dose is given after a wide oscillation of temperature, and results in a fall of temperature with smaller oscillations. Without the guidance of the opsonic index, it is possible to judge by the temperature when to give the next injection. The indication is shown by a sudden rise in temperature, or an increase in the oscillation. If the original dose is followed by a decided reaction, the same dose should be repeated, but if, as in this case, a fall of temperature occurs (as on March 19th), an increased dose should be given on the next occasion (March 26th).

The above patient had chronic pulmonary tuberculosis of one year's duration, with a previous history of pneumonia at the age of 18, and bronchitis on many occasions since. His illness commenced with hæmoptysis. He has a bad family history, the father and two sisters dying of consumption.

Both lungs were affected, and there were abundant crepitant râles heard over the whole left lung and the upper part of the right. The pneumococcic injections were increased to 20 millions. Many of the crepitant râles had disappeared on discharge.

CASE 3. EFFECTS ON TEMPERATURE OF A DOSE OF PNEUMOCOCCUS VACCINE.

In the cases of pulmonary tuberculosis under investigation Inman adopts the following classification, which is also in vogue at Brompton Hospital :—

Group 1.—Resting febrile, cases with fever at rest in bed.

Group 2.—Ambulant febrile, resting afebrile.

Group 3.—Ambulant afebrile.

Group 4.—Working afebrile.

Each of these classes is divided for our present purposes into three sub-divisions, following again Inman's lead :—

(*a*) Infection by tubercle bacilli and secondary organisms.

(*b*) Infection by tubercle bacilli alone.

(*c*) Infection by secondary organisms alone.

The cases I have to present vary greatly in the results of treatment. Necessarily my cases fall under sub-divisions (*a*) and (*c*), and these again under febrile and afebrile groups.

The most successful are those of sub-division (*c*) falling under groups 3 and 4. [Quiescent tuberculosis with infection by secondary organisms alone.]

Others will be found in sub-divisions (*a*) and (*c*), but with febrile disturbance, groups 1 and 2, due in some cases to the double infection of tubercle and secondary organism, and in others to the preponderating influence of the secondary pathogenic bacteria.

### CASES.

I.—Of active and progressive pulmonary tuberculosis with T.B. in the sputum complicated by secondary infection.

Cases 3 and 4.—Effect of *Pneumococcus* Vaccine.

II.—Latent or quiescent pulmonary tuberculosis without T.B. in the sputum, in which the symptoms and signs indicated the presence of a secondary infection.

Cases 5 and 6.—Effect of *Pneumococcus* Vaccine.

Case 7.—Effect of *Micrococcus Catarrhalis* Vaccine.

Case 8.—Effect of *Bacillus Friedländer*.

Case 4. Effects of Pneumococcus Vaccine.

Case 4.—Chronic pulmonary tuberculosis, asthma, pneumococcus infection. H. T., æt. 39, clerk. Admitted to Brompton Hospital Aug. 28th, 1912. Discharged Feb. 8th, 1913. Wife has pulmonary tuberculosis, with asthma. The patient had a profuse hæmoptysis nine years ago, and again two months ago. Suffered on admission from cough, expectoration and paroxysmal dyspnœa. T. B. were found on July 30th and Aug. 29th.

The physical signs shown in the accompanying diagram were those of consolidation of the upper part of the right lung, with weak breath sounds, increased vocal fremitus and voice resonance, with coarse inspiratory crepitant sounds over the dull area.

The patient was treated first with tuberculin without improvement.

A culture was made from the sputum, and a diplococcus pneumoniæ (Fraenkel's) found. A vaccine was prepared and injected.

Result : Much improvement in dyspnœa, less cough and expectoration, diminution of crepitant râles, and complete loss of paroxysmal asthmatic attacks. Gain in weight from 10 stone 1 lb. to 11 stone 3 lb.

CASE 4. EFFECTS OF PNEUMOCOCCUS VACCINE.

Case 5. Effects of Pneumococcus Vaccine.

Case 5.—D. M. G., aged 20, single, living at home. General debility, loss of flesh and cough since July, 1910. Admitted to Brompton Hospital 13th Feb., 1911. Previously an out-patient since July, 1910. Symptoms more active since Sept., 1910.

Two sisters affected with pulmonary tuberculosis; one died of tuberculous pleurisy in July, 1910; and one sister has chronic pulmonary tuberculosis.

T. B. found in sputum on several occasions, Jan. and Feb., 1911. March 5th, cough worse. Signs of consolidation at left base.

The physical signs gradually improved under vaccine treatment, while the temperature gradually fell to normal.

The tubular breathing at base had disappeared by May 9th, though dulness and weak breathing with a few bubbling râles persisted. No adventitious sounds heard in right lung. Gain in weight from 8 stone 12 lb. to 9 stone $6\frac{1}{4}$ lb. Cough and expectoration much diminished.

Continued well for six months, since which time she has been lost sight of.

CASE 5. EFFECTS OF PNEUMOCOCCUS VACCINE.

Case 5.

A chart is appended showing an elaborate investigation in this case by Dr. Inman of the opsonic index to tubercle O, the temperature T, the opsonic index to the pneumococcus P, and to a staphylococcus S, all taken at intervals during twenty-four hours (taken from Inman's paper referred to above). It will be observed that the opsonic index to tubercle is not in inverse ratio to the temperature, and it may be concluded that the raised temperature is mainly due to the mixed infection.

CASE 6. CHRONIC PULMONARY TUBERCULOSIS. PNEUMOCOCCIC INFECTION.

CASE 6.—W. B., aged 30, labourer, was admitted to the Brompton Hospital on Nov. 10th, 1913. He contracted a cold in the previous February, with cough and expectoration, and this continued during the whole summer. The family history was good, with exception of one brother who died of pleurisy at the age of 23. The patient's wife developed consumption, and he nursed her from July, 1912, to August, 1913, when she died.

On admission, it is noted that the duration of illness was ten months, and that T. B.'s were found in the sputum on Nov. 12th, 1913. The physical signs were those of double apical consolidation, greater at the right apex. But there were abundant crepitant signs over the whole of both lungs, greatly exaggerated at the bases behind. The diagnosis made was of chronic pulmonary tuberculosis, with secondary catarrhal infection.

An examination of sputum gave an abundant culture of pneumococci only, without any mixed infection. A vaccine was prepared.

The difficulty with this patient, as in most hospital patients, is that the process of vaccine treatment is prolonged, and though the patient improved and felt better after each injection, the time of treatment in hospital is of necessity too short to produce the full effects.

The result of treatment was a diminution of basic râles, but a good deal of rhonchus and sibilus on discharge, instead of the crepitant râles. Considerable gain in weight ensued from 7 stone 9 lbs. to 8 stone and ½ lb., the general condition proportionately improved.

The temperature chart illustrates the effect of vaccine treatment in steadying the temperature.

CASE 6. CHRONIC PULMONARY TUBERCULOSIS. PNEUMOCOCCIC INFECTION.

### Effect of Micrococcus Catarrhalis Vaccine.

Case 7.—Case of Chronic Pulmonary tuberculosis (quiescent), with secondary infection of micrococcus catarrhalis.

R.W., æt. 26, cook-housekeeper, admitted to Brompton Hospital Jan. 20th, 1914; discharged herself April 11th, 1914. Family history good, parents, brothers and sisters alive and well.

The patient has suffered from shortness of breath for two years, but has been much better last summer, until Nov., 1913, when she was taken ill with an acute catarrhal attack, with cough, short breath, night sweats and profuse expectoration. Some loss of weight succeeded the attack. On Dec. 24th, 1913, acute broncho-pneumonia supervened, and she has been in bed since. The sputum has been examined many times for T. B., but none found. I saw her first in consultation with Dr. Hamilton Bland, and advised an investigation of sputum and the preparation of a vaccine. The micrococcus catarrhalis was found in abundance, and the administration of a vaccine produced immediate improvement.

The physical signs of disease were those of induration of both apices, with abundant catarrhal signs (*i.e.* double crepitations) heard over the rest of both lungs.

The patient did not do very well at home, and at request she was admitted to my ward. On admission no T. B. were found by the antiformin method. The opsonic index to tubercle was 1.62 before exercise and 1.48 after exercise (*i.e.* strongly positive). The complement fixation test gave a negative result. The Von Pirquet cutaneous test also gave a negative reaction.

An examination of sputum for catarrhal organisms produced an abundant growth of the micrococcus catarrhalis, with a smaller culture of a streptococcus. A vaccine was prepared from the sub-culture of M. Catarrhalis.

The effects of the injection of vaccine have on every occasion been immediate and beneficial. So marked has this been, that the patient herself showed a craving for each dose as it became due. On discharge the note is:—There is now no sputum. The crepitations have entirely disappeared from both bases, and only sparse crepitation sounds are now heard at the apices of upper lobes. The patient has gained 10 lb. in weight.

CASE 7.

1. Broncho cavernous breathing, fading towards middle lobe, where the respiration is bronchial.

At the base of the upper lobe impaired resonance, v. vibration, and v. resonance.

2. Fine, small and sharp inspiratory crepitations.

3. Prolonged expiration. All over right back a sparse crepitation with inspiration and expiration.

4. A few crepitant sounds, with inspiration and expiration all over back of left lung.

CASE 7. EFFECT OF MICROCOCCUS CATARRHALIS VACCINE.

CASE 8.—S. S., aged 65, farm labourer, admitted to Brompton Hospital Nov. 3rd, 1911. *Diagnosis probably old P. T., chronic pneumonia, bronchiectasis.*

In August, 1910, a severe attack of pneumonia; ill four weeks, short breath, cough and expectoration ever since. The expectoration has increased often a cupful in the night, with some fœtor; increased by change of posture; often much morning expectoration.

On admission presents much emaciation, cough, dyspnœa on exertion, with free expectoration. Movement of chest and expansion diminished. Well-marked induration of right upper lobe, with dulness, increased v. vibration and coarse broncho cavernous breathing, and increased voice respiration. One or two crepitant râles are present on inspiration. At the back of the right lung there are similar signs with cavernous respiration at apices of upper and lower lobes. No crepitant sounds or adventitious sounds except at the base of the right lower lobe, from angle of scapula downwards. Here there are numerous large inspiratory crepitant râles. The left apex presents signs of consolidation, but no superadded sounds. The fingers are clubbed.

DIAGNOSIS.—Chronic pneumonia, possibly of tuberculous origin, on account of the apical disease, with dilated bronchi. No tubercle bacilli were found on four examinations, including the antiformin method. On examination of the sputum for other organisms almost a pure culture of the bacillus Friedländer was discovered, and a vaccine made. A few pneumo-streptococci were also found.

Soon after admission on Nov. 3rd there was slight hæmoptysis to the extent of ʒ i, and on Nov. 11th about ʒ ii. The amount of sputum (measured) varied from 26 oz. per diem at most to 15 oz.

Vaccine treatment (Friedländer's bacillus) was commenced on Nov. 25th, with three millions; on Nov. 30th 6 millions; and on Dec. 5th 10 millions. The first febrile reaction succeeded this dose, and after several other injections a febrile reaction occurred. Increasing doses of vaccine were given every few days. Last dose of vaccine 25 millions on March 2nd. Sputum diminished from a maximum of 25 oz. to nil. The patient left with a small and variable amount of expectoration not exceeding ʒ i, and with diminished catarrhal sounds and signs.

SUBSEQUENT HISTORY OF THE CASE.—Gain in weight $25\frac{1}{2}$ lb., from 8 stone $5\frac{1}{2}$ lb. to 10 stone 3 lb.; a few crepitations at right base only; improvement in strength; five months' treatment.

It is obvious from the cases presented that the results of treatment by vaccine are variable. My conclusions, as I have stated, are that the best results are obtained from vaccine treatment when the primary tuberculous infection is not active and the secondary symptoms of bronchial catarrh or asthma are prominent. We meet with many such cases, and the symptoms are very chronic and intractable.

I have personally obtained the best results in cases of secondary infection by the *Micrococcus catarrhalis, Fraenkel's diplococcus pneumoniæ* and the *Friedländer bacillus.* A few of the cases have shown such marked and immediate improvement, that it appears to me to fully justify the vaccine treatment. A large number of cases of pulmonary tuberculosis are infected with an organism known for the present as a pneumo-streptococcus, in reality a streptococcus. I have rarely obtained more than temporary relief in such cases, and the results have been disappointing.

I trust that the narration of my own experience, with its successes and failures, may be of assistance to those who are conducting similar investigations, and may help to elucidate a subject which to me is one of supreme interest.

## Figures and Tables

**Case 1. f1:**
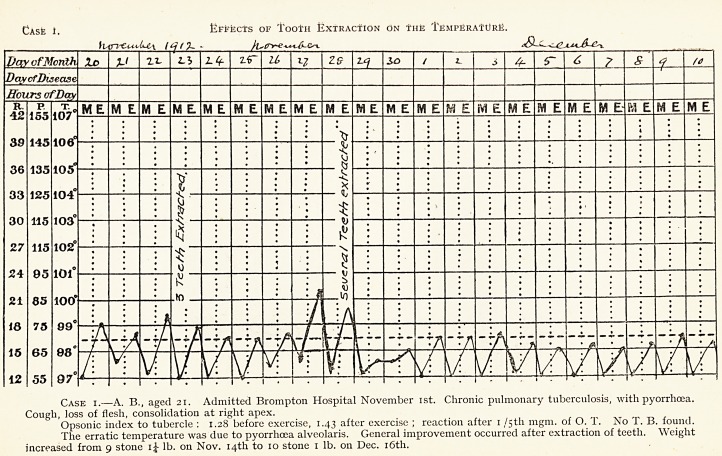


**Case 2. f2:**
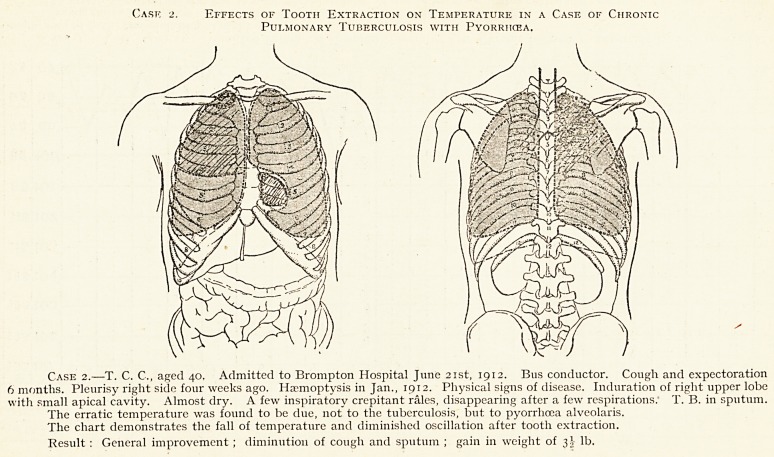


**Case 2. f3:**
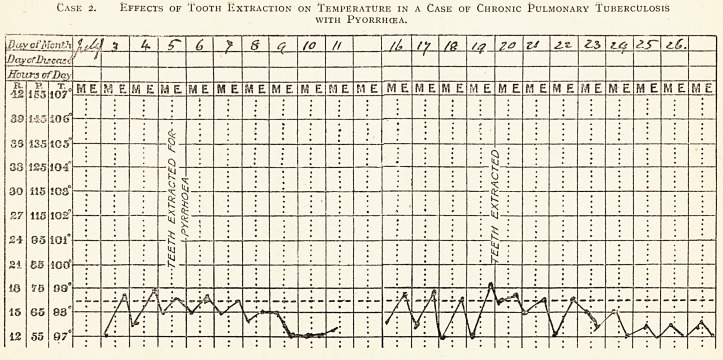


**Case 3. f4:**
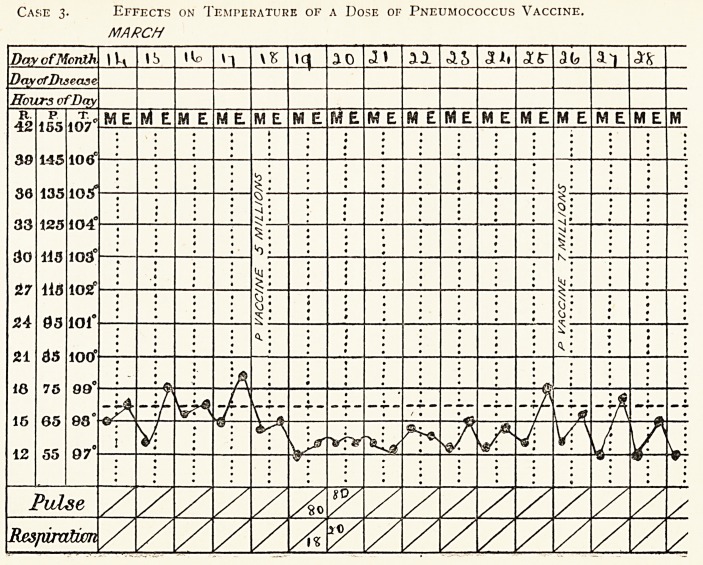


**Case 4. f5:**
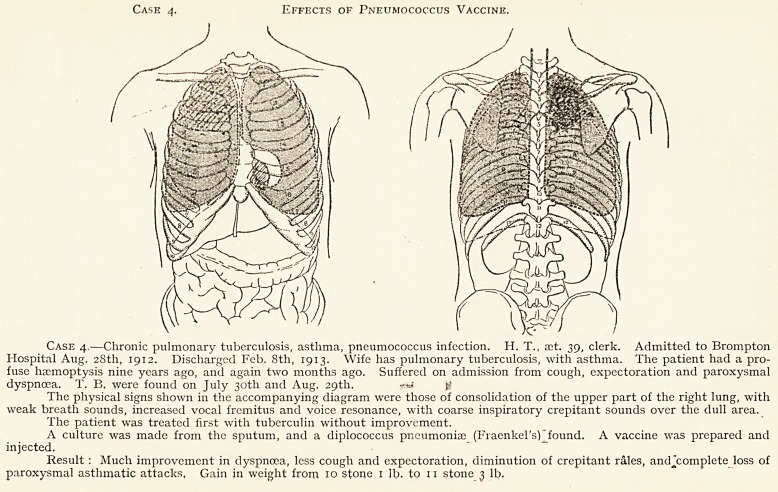


**Case 4. f6:**
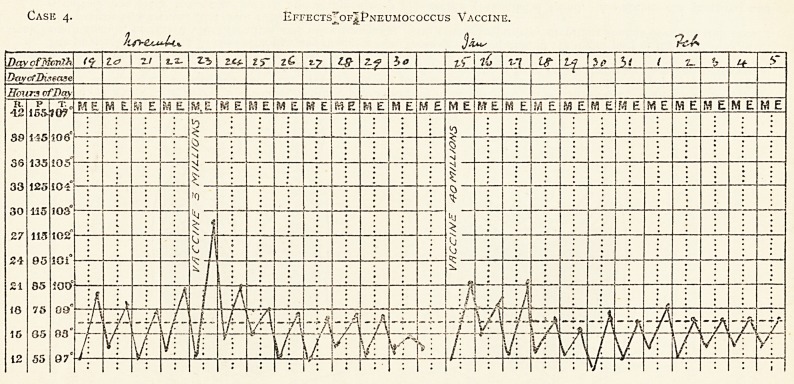


**Case 5. f7:**
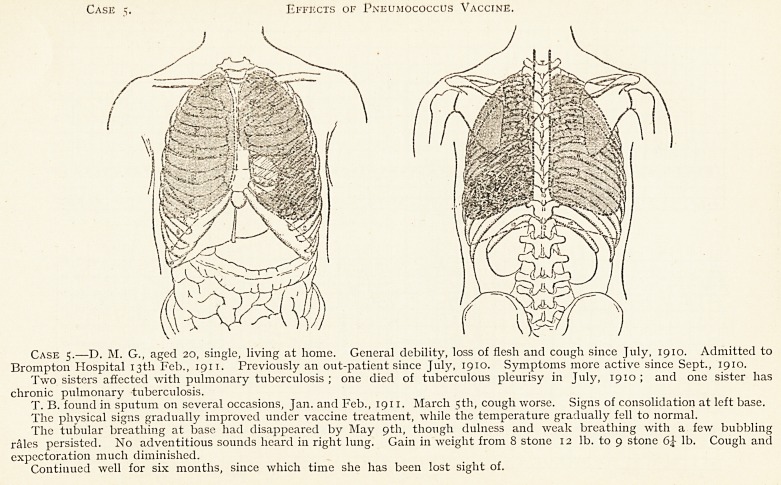


**Case 5. f8:**
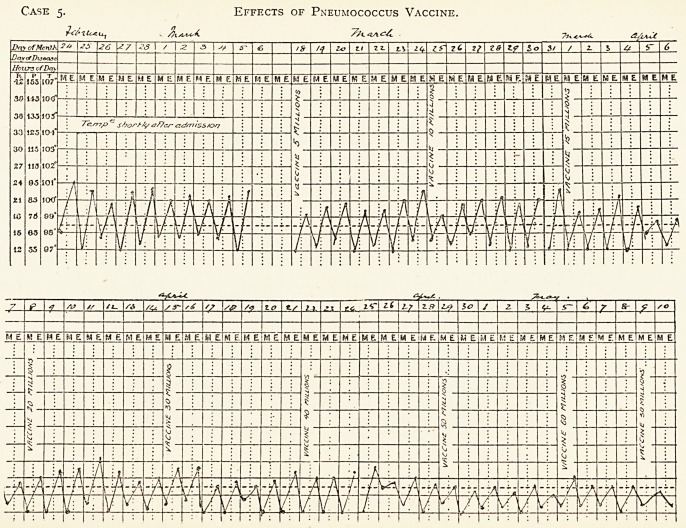


**Case 5. f9:**
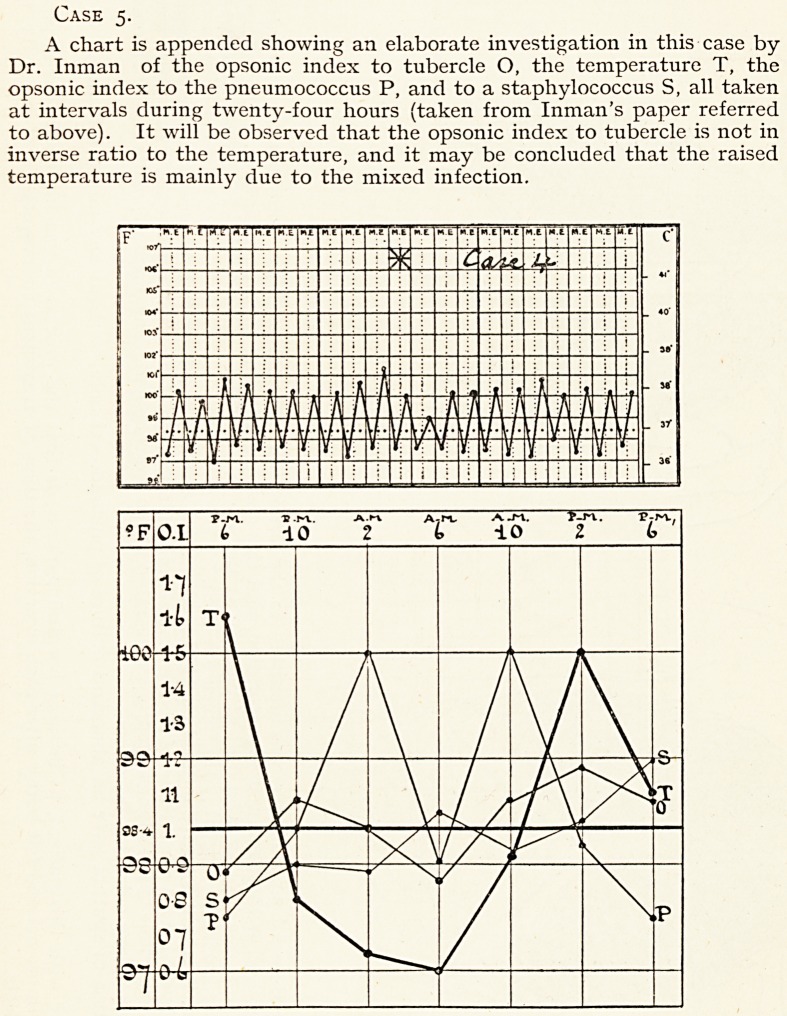


**Case 6. f10:**
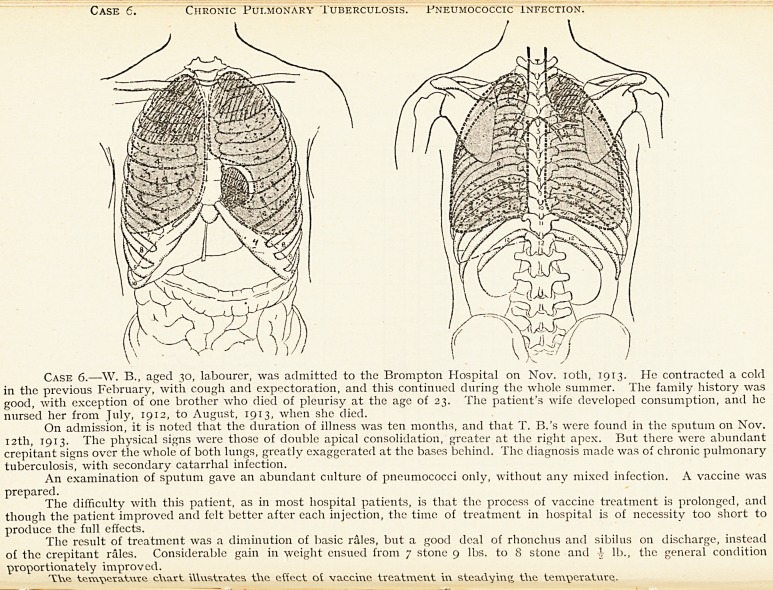


**Case 6. f11:**
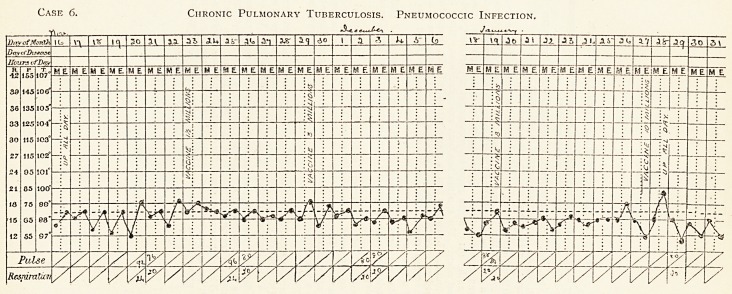


**Case 7. f12:**
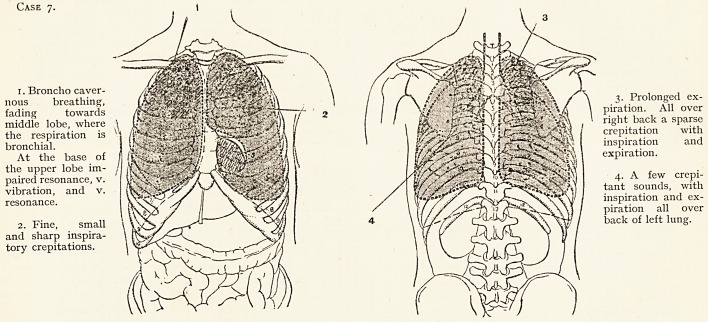


**Case 7. f13:**
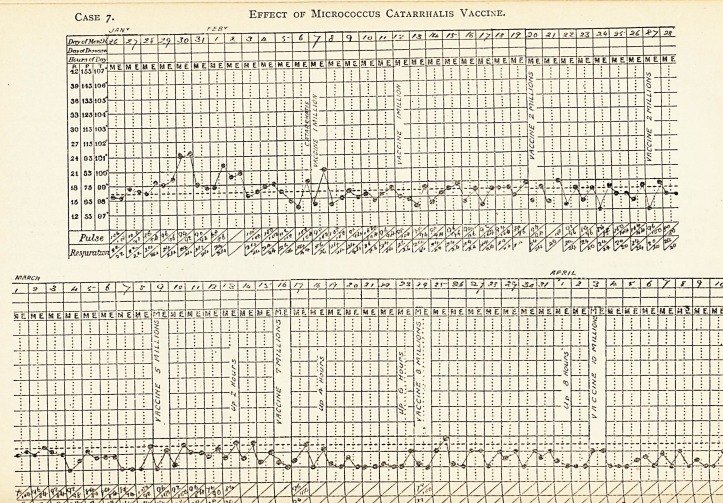


**Case 8. f14:**